# T-bet is essential for Th1-mediated, but not Th17-mediated, CNS autoimmune disease

**DOI:** 10.1002/eji.201343689

**Published:** 2013-07-23

**Authors:** Richard A O'Connor, Helen Cambrook, Katja Huettner, Stephen M Anderton

**Affiliations:** Medical Research Council Centre for Inflammation Research, Centre for Multiple Sclerosis Research and Centre for Immunity Infection and Evolution, Queen's Medical Research Institute, University of EdinburghEdinburgh, UK

**Keywords:** EAE, T-bet, T cells, Th1, Th17

## Abstract

T cells that produce both IL-17 and IFN-γ, and co-express ROR-γt and T-bet, are often found at sites of autoimmune inflammation. However, it is unknown whether this co-expression of T-bet with ROR-γt is a prerequisite for immunopathology. We show here that T-bet is not required for the development of Th17-driven experimental autoimmune encephalomyelitis (EAE). The disease was not impaired in T-bet^−/−^ mice and was associated with low IFN-γ production and elevated IL-17 production among central nervous system (CNS) infiltrating CD4^+^ T cells. T-bet^−/−^ Th17 cells generated in the presence of IL-6/TGF-β/IL-1 and IL-23 produced GM-CSF and high levels of IL-17 and induced disease upon transfer to naïve mice. Unlike their WT counterparts, these T-bet^−/–^ Th17 cells did not exhibit an IL-17→IFN-γ switch upon reencounter with antigen in the CNS, indicating that this functional change is not critical to disease development. In contrast, T-bet was absolutely required for the pathogenicity of myelin-responsive Th1 cells. T-bet-deficient Th1 cells failed to accumulate in the CNS upon transfer, despite being able to produce GM-CSF. Therefore, T-bet is essential for establishing Th1-mediated inflammation but is not required to drive IL-23-induced GM-CSF production, or Th17-mediated autoimmune inflammation.

## Introduction

An illuminating series of studies over the past 5 years has led to the appreciation that, far from being members of categorically fixed subsets, CD4^+^ T cells can switch their expression of transcription factors and therefore downstream effector functions including cytokines, dependent on the inflammatory environment they find themselves in 1. An excellent pathological example of this T-cell plasticity comes from the study of the CD4^+^ T-cell population that drives experimental autoimmune encephalomyelitis (EAE) in the mouse central nervous system (CNS). Observations that this cardinal “Th1-driven” disease was independent of either IFN-γ or IL-12 2,3, led to the appreciation of the indispensible role of IL-23 4 and, ultimately, to the characterization of the IL-17-producing, ROR-γt-expressing, Th17 subset and its importance in EAE 5. This fits with data from cytokine-deficient mice revealing that, as well as IL-23 and ROR-γt, both IL-6 and IL-1 (important factors in Th17 differentiation) are indispensible for EAE 5,6. Nevertheless, interrogation of the T-cell infiltrate in the CNS commonly reveals strong production of IFN-γ and expression of the Th1-master regulator T-bet. Moreover, T-bet has been reported to be essential for EAE 7, even in passive transfer models involving administration of myelin-reactive Th17 cells 8. This paradox has been resolved by elegant fate-mapping studies that revealed that the majority IFN-γ^+^ and T-bet^+^ T cells in the CNS have previously expressed, but since extinguished, IL-17 9,10.

The reported requirement for T-bet in EAE, whether driven by Th1 or Th17 populations, promotes this transcription factor as a potential alternative therapeutic target, beyond individual T-cell-derived cytokines. In light of this, we sought to better understand the role of T-bet in EAE, starting with the hypothesis that it would have a key role in promoting or maintaining GM-CSF, which has recently come to the fore as an essential product of pathogenic T cells 11,12. We report that, while T-bet is required for the IL-17→IFN-γ switch, this is not required for EAE development.

## Results and discussion

### T-bet is not required for the development of autoimmune CNS inflammation

**Figure 1:**
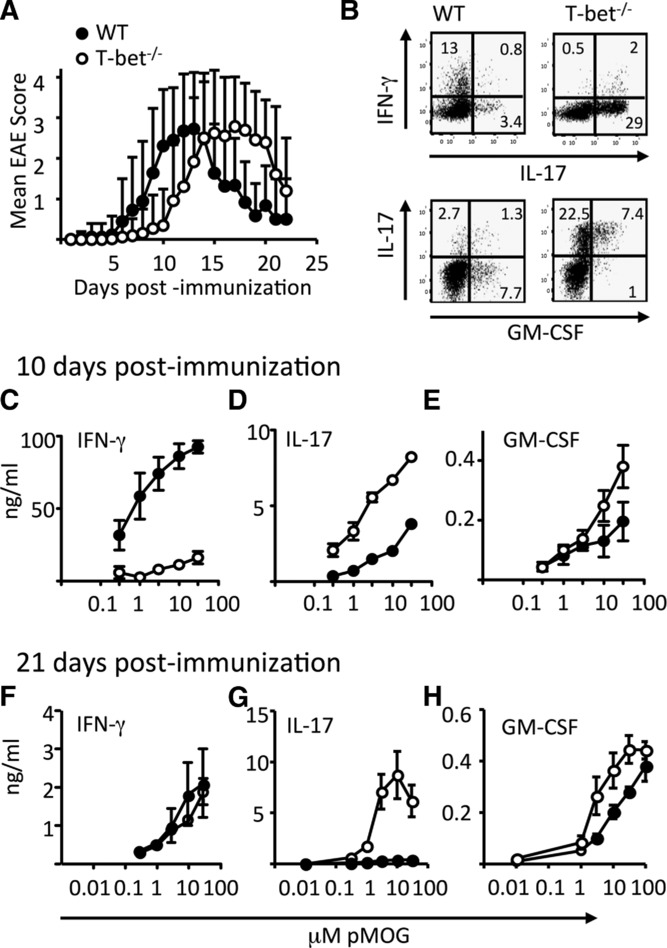
T-bet is not required for the development of EAE. EAE was induced in WT and T-bet^−/−^ mice by immunization with pMOG in CFA. (A) Clinical course of disease in WT (closed symbols) and T-bet^−/−^ (open symbols) mice. Clinical scores are pooled from five independent experiments and represent a total of 36 WT and 65 T-bet^−/−^ mice. (B) pMOG-stimulated cytokine production by CNS-infiltrating CD4^+^ T cells retrieved at day 14 postimmunization. (C–H) pMOG-stimulated production of (C, F) IFN-γ (D and G) IL-17, and (E and F) GM-CSF by splenocytes from WT (closed symbols) and T-bet^−/−^ (open symbols) mice at day 10 and 21 postimmunization. Results shown are from one experiment and are representative of three independent experiments with five to six mice per group per experiment. Data are shown as mean ± SEM. (C and D) *p* < 0.001 and (E and G) *p* < 0.05, two-way ANOVA with Bonferroni posttest.

T-bet^−/−^ mice developed EAE after immunization with the myelin oligodendrocyte glycoprotein 35–55 peptide (pMOG). While there was consistently a delay in disease onset in the absence of T-bet (Fig. 1A), the peak severity of disease did not differ significantly between WT and T-bet^−/−^ mice. IFN-γ was reduced (but not totally absent) in T-bet^−/−^ T cells in the CNS (Fig. 1B). In contrast, IL-17 production was markedly elevated in the absence of T-bet (Fig. 1B). As described in earlier fate-mapping studies 9,10, IFN-γ^+^ CNS T cells were almost entirely IL-17-negative by the peak of disease in WT mice. In contrast, the fewer IFN-γ^+^ T cells in T-bet^−/−^ mice were mainly also IL-17^+^. The frequencies of GM-CSF^+^ CD4^+^ cells in the CNS did not differ between WT and T-bet^−/−^ mice (Fig. 1B, lower panels).

Recall responses to pMOG (Fig. 1C–H) showed reduced (but not totally absent) IFN-γ, but elevated IL-17 from T-bet^−/−^ splenocytes. Dynamic changes in levels of IFN-γ production were evident over time in WT mice, whereas IFN-γ production remained at a low level in T-bet^−/−^ mice. Thus, at 10 days after priming, IFN-γ production was high in WT, but greatly impaired in T-bet^−/−^ splenocytes (Fig. 1C), but by 21 days WT and T-bet^−/−^ splenocytes produced equivalent (low) levels of IFN-γ (Fig. 1F). Again, GM-CSF production was not impaired in T-bet^−/−^ mice (Fig. 1E and H).

We conclude that encephalitogenicity does not require T-bet and that this might reflect maintained GM-CSF and/or enhanced IL-17 production in its absence.

### Pathogenicity of autoreactive Th1 cells is T-bet dependent

Th1 cells, generated from naive T cells that have not passed through the IL-17-producing stage that occurs in vivo after immunization, can induce EAE and also produce GM-CSF 11,12. We therefore asked whether T-bet^−/−^ T cells exposed to Th1-promoting conditions (IL-12 and IL-18) would maintain GM-CSF production and pathogenic function.

**Figure 2:**
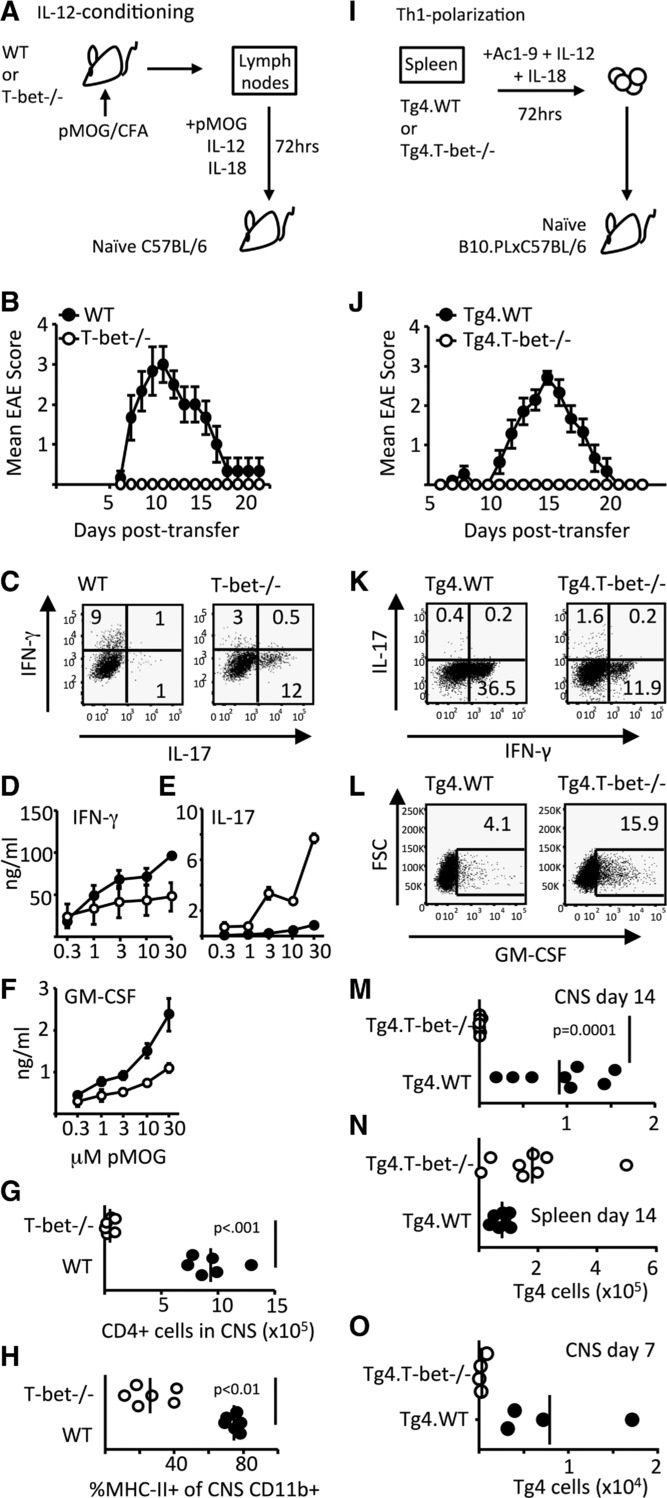
Pathogenicity of Th1 cells is T-bet dependent. (A–H) EAE was induced in C57BL/6 mice by passive transfer of WT or T-bet^−/−^ IL-12-conditioned pMOG-responsive cells. (B) Clinical course of disease is shown. (C) IFN-γ and IL-17-producing capacity of cells on the day of transfer was assessed by flow cytometry. (D–H) Cohorts of mice were sacrificed at 12 days posttransfer to assess cytokine production and CNS inflammation. The production of (D) IFN-γ, (E) IL-17, and (F) GM-CSF by pMOG-stimulated splenocytes is shown. (E) *p* < 0.001 and (F) *p* < 0.05, two-way ANOVA with Bonferroni posttest. (G) The numbers of CD4^+^ T cells in the CNS and (H) the percentage of CNS CD11b^+^ cells expressing MHC class II in recipients of WT (closed symbols) and T-bet^−/−^ cells (open symbols) are shown. (A–H) All data shown are from one experiment representative of two independent experiments with six mice per group per experiment. (I–O) EAE was induced in B10.PLxC57BL/6 mice by passive transfer of Th1-polarized Tg4.WT or Tg4.T-bet^−/−^ T cells. (J) Clinical course of disease is shown. Results shown are from one experiment representative of three independent experiments with eight to nine mice per group per experiment. Data are shown as mean ± SEM. (K) IFN-γ and IL-17 and (L) GM-CSF producing capacity of cells on the day of transfer are shown. (M–O) The numbers of transferred Tg4 cells retrieved from (M) the CNS and (N) spleen at 14 days posttransfer or (O) the CNS at 7 days posttransfer are shown.

We first generated T cells for passive transfer from LNs of H-2^b^ mice that have been immunized with pMOG (Fig. 2A). WT IL-12-conditioned cells transferred robust EAE, whereas T-bet^−/−^ cells did not transfer disease (Fig. 2B). At the time of their transfer, WT IL-12-conditioned cells produced IFN-γ with very few staining for IL-17. In their T-bet^−/−^ counterparts, this pattern was reversed (Fig. 2C). The transferred T-bet^−/−^ cells seemed able to persist in the spleen as indicated by a strong production of IL-17 upon pMOG recall (while IFN-γ and GM-CSF production were reduced, but not absent), compared to spleens of mice receiving WT cells (Fig. 2D–F). The failure of IL-12-conditioned T-bet^−/−^ cells to drive EAE correlated with an inability to migrate to (or expand in) the CNS as evidenced by poor representation of CD4^+^ or MHC-II^+^ CD11b^+^ cells in the CNS (Fig. 2G and H).

Using a second system, in which Th1 cells are generated from naïve myelin basic protein (MBP) responsive Tg4 TCR transgenic T cells by MBP peptide stimulation in the presence of IL-12 and IL-18 (Fig. 2I), we also found that passive EAE was dependent on expression of T-bet in the transferred T cells (Fig. 2J). At the time of transfer, the frequency of IFN-γ^+^ cells was reduced (but not absent) in Tg4.Tbet^−/−^ Th1 cells (Fig. 2K). The lack of a profound increase in IL-17 expression by these cells (Fig. 2K) differed from the elevated IL-17 that was seen in pMOG-immunized T-bet^−/−^ cells (Fig. 1B, D, and G), even after in vitro exposure to IL-12 (Fig. 2C). This difference is likely to reflect exposure to IL-17-promoting factors (IL-1, IL-6, and IL-23) during in vivo priming of T-bet^−/−^ mice, but not during the in vitro polarization of Th1 cells from naive Tg4.T-bet^−/−^ cells. GM-CSF production was not impaired in Tg4.Tbet^−/−^ Th1 cells (Fig. 2L). Again, lack of disease reflected an inability of the transferred Tg4.Tbet^−/−^ Th1 cells to accumulate in the CNS and their retention in the spleen (Fig. 2M–O).

### Pathogenicity of autoreactive Th17 cells is T-bet independent

**Figure 3:**
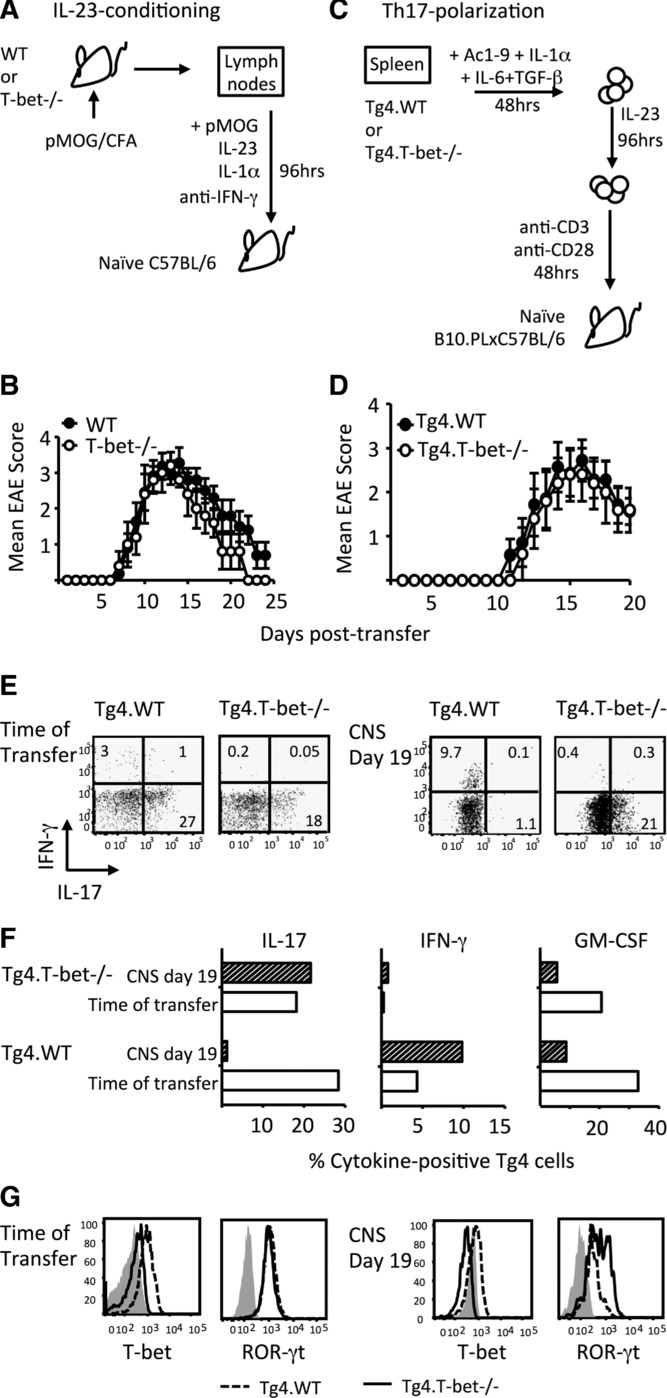
Pathogenicity of Th17 cells is T-bet independent. (A and B) EAE was induced in C57BL/6 mice by passive transfer of WT or T-bet^−/−^ IL-23-conditioned cells derived from the draining LNs of pMOG-immunized mice. (B) Clinical course of disease is shown. Results are from one experiment representative of two independent experiments with *n* = 5 per group per experiment. Data are shown as mean ±SEM. (C–G) EAE was induced in B10.PLxC57BL/6 mice by passive transfer of Th17-polarized Tg4.WT or Tg4.T-bet^−/−^ T cells. (D) Clinical course of disease is shown. Results are from one experiment representative of two independent experiments with 5–11 mice per group per experiment. Data are shown as mean ± SEM. (E–G) Flow cytometric analysis of cytokine production and transcription factor expression in Tg4.WT and Tg4.T-bet^−/−^ Th17 cells at the time of transfer and after retrieval from the inflamed CNS at 19 days posttransfer. (E) IL-17 versus IFN-γ production, (F) individual cytokines, and (G) T-bet and ROR-γt (G: gray histograms show isotype control staining).

The phenotype of the T-bet^−/−^ CNS T cells in active EAE (Fig. 1B) suggested a population of stable Th17 cells. We therefore generated Th17-polarized cells and tested their requirement for T-bet to induce EAE (Fig. 3). IL-23-conditioned T cells from pMOG-primed LNs of T-bet^−/−^ mice had high-IL-17 production and both WT and T-bet^−/−^ cultures showed strong expression of GM-CSF at the time of transfer (data not shown) and were equally encephalitogenic, providing indistinguishable clinical courses (Fig. 3B). We also generated Th17 cells from naïve Tg4 T cells (Fig. 3C) and again found that WT and T-bet^−/−^ Tg4 cells were equally encephalitogenic (Fig. 3D). Consistent with the paradigm in which IL-17^+^ T cells begin to express T-bet, extinguish IL-17, and switch-on IFN-γ production over the course of EAE, Tg4.WT Th17 cells had limited IFN-γ production at the time of transfer, but had gained this function and were IL-17-negative when retrieved from the CNS (Fig. 3E and F). This shift in function did not occur in Tg4.T-bet^−/−^ Th17 cells. Their IL-17 production was maintained in the CNS, with no evidence for IFN-γ production (Fig. 3E and F).

Not only was T-bet required for the gain of IFN-γ production, it was also necessary for the loss of IL-17 production by cells retrieved from the CNS (Fig. 3E and F). This is consistent with the CNS CD4^+^ cytokine profiles displayed by WT versus T-bet^−/−^ mice during pMOG-induced active EAE (Fig. 1B). There are two possible T-cell-intrinsic mechanisms for the loss of IL-17 production when T-bet is present: (i) via the interaction of T-bet with runx1 preventing runx1-mediated transactivation of the *Rorc* promoter 13; (ii) by boosting IFN-γ production which in turn inhibits IL-17 production 14. Fitting with either scenario, whereas ROR-γt expression was lost in WT Th17 cells retrieved from the CNS, at least some of their T-bet^−/−^ counterparts maintained ROR-γt to pretransfer levels (Fig. 3G).

We have found no evidence for an impairment of proliferative capacity in T-bet^−/−^ T cells (data not shown), so we believe that the absence of transferred T-bet^−/−^ Th1 cells from the CNS most likely reflects a failure in migration. Expression of CCR6, which has been proposed to facilitate Th17 cell entry into the CNS (reviewed in 15), was not affected by the lack of T-bet in Tg4 Th17 cells (data not shown). As would be expected, T-bet deficiency did impair expression of Th1-associated CXCR3 in Th1-polarized Tg4 cells (data not shown). However, we cannot currently conclude that this accounts for the inability of T-bet^−/−^ IL-12-conditioned or Th1 cells to enter the CNS, because there is no consensus on the importance of CXCR3 expression for Th1-induced EAE 15. The molecular basis for the failure of T-bet^−/−^ Th1 cells to accumulate in the CNS therefore remains to be provided.

Our data differ significantly from those recently reported by Duhen et al. 16. That report reasserted that mice with global T-bet deficiency are resistant to EAE and implied possible necessary roles for T-bet expression by non-T cells. We show that this is not the case. Duhen et al. 16 used mice in which T-bet-deficiency was controlled by the CD4 promoter and showed the same delayed course of EAE that we see here with T-bet^−/−^ mice. That report also concluded that the ability to produce IFN-γ co-associated with pathogenic function of Th17 cells, even in the absence of T-bet. Here, we show that, in the absence of T-bet, Th17 cells can drive EAE without the development of IFN-γ production. Subtle differences in approach between the two studies might account for these discrepancies. For example, Duhen et al. 16 used chronic in vitro stimulation with IL-23 to engender IFN-γ production by T-bet^−/−^ Th17 cells. In contrast, our Th17 cells were wholly capable of pathogenic function without such extended in vitro manipulation. However, the study by Duhen et al. does concur with ours in the conclusion that T-bet is not critically involved in GM-CSF production by pathogenic T cells.

### Concluding remarks

There is an absolute requirement for the putative pathogenic T cell in EAE to be sensitive to IL-6, IL-23, and IL-1 and to produce GM-CSF. Our data, combined with those of others 11,12, indicate that there is no gross deficiency in these critical features in the absence of T-bet. Based on this, Th17-driven inflammation should be unimpaired in T-bet^−/−^ mice. This is what we find here. While increased T-bet expression is certainly a characteristic of pathogenic Th17 cells 17, we have shown definitively that it is entirely nonessential for EAE development and that Th17 cells can induce inflammatory pathology without co-opting those elements of the Th1 transcriptional program under the control of T-bet. Thus, T-bet may be a less attractive therapeutic target than it has previously appeared.

## Materials and methods

### Mice, antigens, and tissue culture medium

C57BL/6, Tg4.CD45.1 (Tg4.WT) 18, T-bet^−/−^ (obtained from The Jackson Laboratory), and B10.PLxC56BL/6 mice were bred under specific pathogen-free conditions at the University of Edinburgh. T-bet^−/−^ mice were crossed with Tg4.CD45.1 mice to generate Tg4.T-bet^−/−^ mice. All experiments were approved by the University of Edinburgh ethical review committee and were performed in adherence to UK legislation. Mice were screened in-house, using the Jackson Labs protocol to confirm knockout status and independently by Transnetyx Inc. (Cordova, TN, USA). pMOG (MEVGWYRSPFSRVVHLYRNNGK) and the MBP Ac1–9 (Ac-ASQKRPSQR) peptide were synthesized by Cambridge Research Biochemicals (Cleveland, UK). Tissue culture medium was RPMI 1640 medium, supplemented with 2 mM l-glutamine, 100 U/mL penicillin, 100 μg/mL streptomycin, and 5 × 10^−5^ M 2-ME (all from Invitrogen Life Technologies, Paisley, UK), and 10% FCS (Sigma, Poole, UK).

### Active induction of EAE

EAE was induced in H-2^b^ (C57BL/6 and T-bet^−/−^) mice using 100 μg of pMOG_35–55_ peptide emulsified in CFA, as described previously 19. Clinical signs of EAE were assessed daily with the following scoring system: 0, no signs; 1, flaccid tail; 2, impaired righting reflex and/or gait; 3, partial hind limb paralysis; 4, total hind limb paralysis; 5, hind limb paralysis with partial front limb paralysis; 6, moribund or dead. Assessment of CNS (brain and spinal cord) immune cells was as described previously 20.

### Adoptive transfer of EAE

pMOG-reactive cells were generated from draining LN cells 10 days after immunization with pMOG_35–55_ and cultured at 4 × 10^6^ cells/mL in the presence of 10 μg/mL pMOG. pMOG-reactive IL-12 conditioned cells 20, or IL-23 conditioning cells 21, were prepared as previously described.

Tg4 splenocytes were cultured at 4 × 10^6^ cells/mL with 10 μg/mL MBP (Ac1–9). Conditions for Th1 polarization 20 and Th17 polarization 22 were as previously described. In all transfer experiments, 4 × 10^6^ blasts were injected into WT recipients. Clinical signs of EAE were assessed as described above.

### Antibodies and flow cytometry analysis

Cells were stained for flow cytometry using the indicated Abs. Intracellular staining for cytokines and transcription factors was performed as previously described 20. Flow cytometry data were collected on a Fortessa flow cytometer (BD Biosciences) and all data were analyzed using FlowJo software (Tree Star, CA, USA).

### In vitro restimulation and cell culture

A total of 8 × 10^5^ splenocytes per well (in flat-bottom 96-well microtiter plates) were cultured in X-VIVO-15™ tissue culture medium (BioWhittaker, Wokingham, UK) and stimulated with a dose range of pMOG. After 72-h culture, supernatants were removed for determination of cytokine levels by ELISA.

### Statistical analysis

Flow cytometry data were compared by unpaired *t*-test. ELISA data were compared using two-way ANOVA with Bonferroni posttesting.

## Abbreviations

MBP: myelin basic protein

## Supplementary material

As a service to our authors and readers, this journal provides supporting information supplied by the authors. Such materials are peer reviewed and may be re-organized for online delivery, but are not copy-edited or typeset. Technical support issues arising from supporting information (other than missing files) should be addressed to the authors.
