# Supplementary material for: T-bet is essential for Th1-mediated, but not Th17-mediated, CNS autoimmune disease
Source: Eur J Immunol. 2013 Jul 23;43(11):2818–23. doi: 10.1002/eji.201343689 (PMC4068221; doi:10.1002/eji.201343689)
Supplement: Supplementary file 1 [file eji0043-2818-SD1.pdf]

European Journal of Immunology

Supporting Information

for

DOI 10.1002/eji.201343689

Richard A. O'Connor, Helen Cambrook, Katja Huettner and Stephen M. Anderton

T-bet is essential for Th1-mediated, but not Th17-mediated, CNS autoimmune disease

© 2013 The Authors. *European Journal of Immunology* published by WILEY-VCH Verlag GmbH & Co. KGaA Weinheim.

European Journal of Immunology

Peer review correspondence

T-bet is essential for Th1-mediated, but not Th17-mediated, CNS autoimmune disease

Richard A. O'Connor, Helen E. Cambrook, Katja Huettner and Stephen M. Anderton

Corresponding author: Stephen Anderton, Centre for Inflammation Research, Queen's Medical Research Institute

Review Timeline:	Submission date:	8 May 2013
	Editorial decision:	4 June 2013
	Revision received:	14 June 2013
	Accepted:	17 July 2013

Handling Executive Committee member: Prof. Rikard Holmdahl

Please note that the correspondence below does not include the standard editorial instructions regarding preparation and submission of revised manuscripts, only the scientific revisions requested and addressed.

First Editorial Decision – 4 June 2013

Dear Steve,

Apologies for not processing your manuscript ID eji.201343689 entitled "T-bet is essential for Th1-mediated, but not Th17-mediated, autoimmune disease" which you submitted to the European Journal of Immunology as quickly as I'd hoped but I wanted your paper to be reviewed with that of Ben Segal. We now have an editorial decision and the comments of the referees are included at the bottom of this letter.

A revised version of your manuscript that takes into account the comments of the referees will be reconsidered for publication. You should also pay close attention to the editorial comments included below. We will give your revised paper priority.

Please note that submitting a revision of your manuscript does not guarantee eventual acceptance, and that your revision will be re-reviewed by the referee(s) before a decision is rendered.

If the revision of the paper is expected to take more than three months, please inform the editorial office. Revisions taking longer than six months may be assessed by new referees to ensure the relevance and timeliness of the data.

Once again, thank you for submitting your manuscript to European Journal of Immunology and we look forward to receiving your revision.

Best wishes
Cate

On behalf of Prof. Rikard Holmdahl

Dr. Cate Livingstone
Editorial Office
European Journal of Immunology
e-mail: ejied@wiley.com

****

Reviewer: 1
Comments to the Author
O'Connor and colleagues report here that the transcription factor t-bet is dispensable for the initiation of EAE by active immunization and adoptive transfer of TH17 cells, whereas polarized TH1 cells require tbet for their pathogenicity. This is utterly surprising and in contrast to previously published data by others. Overall, this is a very interesting and timely report and I am in favor of its publication in the EJI.

The report has some minor issues which need to be fixed:
1) in the abstract and later on as well, they claim that tbet-deficient T cells fail to induce EAE even though they make gmcsf. However, Figure 2D shows a drastic reduction of GM-CSF levels and thus their statement should be revised.
2) Mechanistically, the authors suggest that migration or reduced expansion into/in the CNS is to blame for the loss of encephalitogenicity of tbet-/- T cells. However, there is no data to support this as the mechanistic underpinning. They found the mice resistant and no signs of inflammation in the CNS (hence no T cells invading the tissues). The reason for this however is unclear. Is that perhaps because i) they do produce less GM-CSF, ii) poor migratory capacity or iii) there is less proliferation in response to MOG? With regard to ii) a migration experiment in vitro could be performed (not critical though). With regard to iii) a simple recall with MOG and an expansion assay (thymidine or cfse) is quickly performed and would shed light onto this. The GM-CSF data (to i) they already have. They are unlikely able to definitively explain as to what tbet-/- T cells fail to transfer EAE, but the discussion should be slightly expanded to cover this.

European Journal of Immunology

Reviewer: 2

Comments to the Author

In this interesting manuscript, O'Connor et al investigate the requirement for T-bet in mediating in experimental autoimmune encephalomyelitis. The key finding of this report is that there were distinct requirements of T-bet for Th1 and Th17 mediated EAE. Whereas T-bet is essential for Th1 cells induced EAE, it is dispensable for Th17 cell mediated EAE. Th17 cells generated in T-bet-/- mice failed to convert to Th1 like cells thus accounting for the lack of IFNg+IL-17+ double positive cells that are often found in EAE brain. This work supports the notion that Th17 cells alone are sufficient to induce EAE and double positive cells are probably not necessary to induce EAE. These new findings raise the doubt whether T-bet, as suggested from previous studies (Bettelli Et al 2004 JEM & Duhen JI 2013), is a viable therapeutic target.

In general, this is an important study that clarifies the role of T-bet in EAE. Below are few comments that should be taken care of.

Comments:

• Although the data presented are clear and important answer to the existing suggestion about T-bet requirement in Th17 mediated EAE, the manuscript stops without addressing the possible reasons for putative discrepancies from the published studies.

• Figure 2: Please show the IFNg ELISA for both WT and T-bet-/- T cells after in vitro conditioning. Also FACS picture for the frequencies of IFNg/IL-17 populations must be shown.

• What is the reason for the inability of T-bet-/- T cells to migrate to the CNS? Is it due to differential chemokine or integrin mediated entry to the CNS?

• Please show isotype control staining for Figure 3G, T-bet staining.

• Page 4: Please remove "…. GM-CSF dependent pathogenic function". There is no indication from this study if GM-CSF is necessary for EAE in this particular setting.

Reviewer: 3

Comments to the Author

In the manuscript by O' Connors et al. "T-bet is essential for Th1-mediated, but not Th17-mediated, autoimmune disease" the authors investigated the role of T-bet in the development of EAE upon the adoptive transfer of Th1 or Th17 cells. The manuscript is of interest, I have few comments that need to be addressed:

Comments:

Title: the authors analysed the role of T-bet in only one model of autoimmune disorder, the title need to be changed.

Fig. 1B: has the cytokine production been induced by antigen specific stimulation? Or polyclonal stimulation? As expected, the authors analyse the ability of CD4+ T cells to produce IL-17 and IFN-g. I'm wondering if other type of lymphocytes are present in CNS at the peak of the disease, i.e CD8 cells. Moreover, if so, are they able to produce IFN-g?

Fig. 2: splenocytes from T-bet-/- mice stimulated in vitro with pMOG are characterized by high IL-17 production (panel C). This is expected because of the data shown by the authors in Fig. 1 and because of Th17 cells, induced in these mice during pMOG/CFA immunization, cannot be adequately polarized in vitro by IL-12 towards the Th1 phenotype (lack of T-bet). I do not understand why these Th17 cells are not able to migrate into the CNS. Is IL-12 suppressing their migratory functions? This point needs an explanation.

First Revision – authors' response – 14 June 2013

Below we address the reviewers' individual questions and detail the changes made to the manuscript (our responses are in red).

Reviewer: 1

The report has some minor issues which need to be fixed:

1) in the abstract and later on as well, they claim that tbet-deficient T cells fail to induce EAE even though they make gmcsf. However, Figure 2D shows a drastic reduction of GM-CSF levels and thus their statement should be revised.

Please see response to 2i) below

2) Mechanistically, the authors suggest that migration or reduced expansion into/in the CNS is to blame for the loss of encephalitogenicity of tbet-/- T cells. However, there is no data to support this as the mechanistic underpinning. They found the mice resistant and no signs of inflammation in the CNS (hence no T cells invading the tissues). The reason for this however is unclear. Is that perhaps because i) they do produce less GM-CSF,

Please note that it is of course only bet-$^{-/-}$ Th1 / IL-12 conditioned cells that fail to induce EAE.

European Journal of Immunology

We have included further data (ICS) showing the ability of the transferred Tg4 Th1 cells to produce GM-CSF on the day of their transfer (new Figure 2L). These show that the frequency of GM-CSF^{+} Th1 cells is not impaired in the absence of T-bet. If anything, Tg4.T-bet$^{-/-}$ cells seem to show higher staining for GM-CSF. Thus, at input, this function does not seem impaired. This makes some sense, given the reported inhibitory effect of IFN-γ on GM-CSF production. The frequency of IFN-γ^{+} cells is lower in the absence of T-bet (new Fig 2J, but note that some cells do still make IFN-γ). It is therefore plausible that this enables the same cell population to be getter GM-CSF producers. However, it is worth noting that through a range of other experiments we have found that within transferred pathogenic T cells the frequency of GM-CSF^{+} donor cells present in the CNS around peak of disease has declined relative to the frequencies at input. Our belief is therefore that the requirement for GM-CSF is transient, promoting the initial establishment of the inflammatory lesion, which might then be sustained with a need for either no, or low, production of GM-CSF from the autoreactive effector T cells. As the reviewer will appreciate, it is extremely challenging o test this because the pre-clinical time-points at which we need to explore this involve very low numbers of infiltrating donor cells. It will take us some time to get to the point where we can assess this definitively.

ii) poor migratory capacity (a migration experiment in vitro could be performed, although not critical

We believe that this is the most likely explanation. This is best exemplified using the Tg4 transfer system because we can accurately trace their fate based on their CD45.1^{-} expression. The original Fig 2I&J (now 2M&N) showed the absence of T-bet$^{-/-}$ cells from CNS at14 days after transfer. We have no added data at a preclinical time (7 days) to show a similar effect (new Fig 2O). We therefore believe that lack of migration is the major factor. We also expand on this point in our response to reviewer 2 below. As would be predicted, T-bet$^{-/-}$ Th1 cells fail to upregulate CXCR3, whereas CCR6 is not impaired in Th17 cells. However, due to the controversies in the field over the importance of these receptors for CNS entry, we feel we cannot definitively conclude that this explains the differences. More exhaustive studies with “add-back” experiments would be required.

Or iii) there is less proliferation in response to MOG? A simple recall with MOG and an expansion assay (thymidine or cfse) is quickly performed and would shed light on this.

We have consistently found no impairment in antigen-driven proliferation of T-bet$^{-/-}$ T cells. This is the case using recall assays after pMOG immunization in the B6 system and also when using MBP-peptide driven activation of naive or Th17 Tg4 T cells. An example of proliferation by WT (filled) and T-bet$^{-/-}$ (open) 7 day primed splenocytes in response to pMOG is shown below. We have chosen not to incorporate these data into the Figures, but have added a sentence to the text to make this point, leading into the discussion of chemokine receptor expression.

European Journal of Immunology

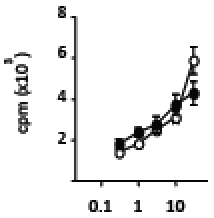

They are unlikely able to definitively explain as to what tbet-/- T cells fail to transfer EAE, but the discussion should be slightly expanded to cover this.

We have discussed the additional new data (GM-CSF production) in the revised text and included a section in which we consider the reason for the failure of T-bet$^{-/-}$ Th1 cells to migrate (CXCR3 versus CCR6). We hope that the modified text incorporates these points sufficiently.

Reviewer: 2

• Although the data presented are clear and important answer to the existing suggestion about T-bet requirement in Th17 mediated EAE, the manuscript stops without addressing the possible reasons for putative discrepancies from the published studies.

The last paragraph prior to our concluding remarks compares our approach with that taken in the most recent paper (Duhen et al.) We feel that to go beyond this currently will enter too far into the realm of speculation. Our present conclusions can only be based on our own data.

• Figure 2: Please show the IFNg ELISA for both WT and T-bet-/- T cells after in vitro conditioning. Also FACS picture for the frequencies of IFNg/IL-17 populations must be shown.

We have added IFN-γ production, to complement IL-17 and GM-CSF post-EAE development (new Fig 2F-H). We have also added ELISA pMMOG recall data at 10 days post-immunization (new Fig 1C-E). These represent the cell populations used to produce the non-pathogenic T cells as in Figure 2B. As can be seen from Fig. 1C-E, IFN-γ is reduced (but not totally absent), IL-17 is enhanced and GM-CSF is enhanced in the T-bet$^{-/-}$ mice.

We have also added IL-17 vs IFN-γ ICS at time of transfer for the B6/MOG system (newFig2C). These show predictable changes in the frequencies of cytokine^{+} cells in the absence of T-bet (IFN-γ lower, IL-17raised). Please note that these experiments were performed some time ago, before we had robust GM---CSF staining working. Thus we do not have GM-CSF data to add for this time point. However, we do have such data for the Tg4 Th1 transfer system,now provided in new Fig. 2K&L. As described in response to reviewer 1, Tg4.T-bet$^{-/-}$ Th1 are not deficient in GM-CSF production at the time of transfer.

European Journal of Immunology

Of interest, although the frequency of IFN-γ$^+$ cells is lower, the concomitant rise in IL17$^+$ cells is less pronounced in the Tg4.T-bet$^{-/-}$ system (which polarizes from naive T cells). This suggests that the enhanced production of IL-17 in the absence of T-bet requires in vivo factors that are not supplied by the in vitro Th1-polarizing protocol (IL-1, IL-6 and IL-23 are obvious candidates). We have added discussion of this point to the revised text.

• What is the reason for the inability of T-bet-/- T cells to migrate to the CNS? Is it due to differential chemokine or integrin mediated entry to the CNS?

Please note that it is only T-bet$^{-/-}$ Th / IL-12 conditioned cells that are impaired in access to the CNS, not Th17 cells.

As would be expected, T-bet$^{-/-}$ Th1 cells fail to upregulate CXCR3 in response to IL---12. This may be too simple an explanation, however, since the literature on the requirement for CXCR3 in EAE is controversial.

Also as would be expected, pathogenic T-bet$^{-/-}$ Th17 cells do not show impaired CCR6 expression.

We have added a section to the discussion to make these points, but we are unable to conclude that absence of CXCR3 is the reason for absence of T-bet$^{-/-}$ Th1 cells from the CNS.

As the reviewer suggests, exhaustive comparison of chemokine receptor and integrin expression, followed by appropriate over---expression experiments to impose CNS access/pathogenic function are required to provide definitive information on this point. The time required for such studies is beyond the current report, which focuses on our novel and important observation that T-bet is dispensable for both “standard” actively induced EAE and Th17-transfer EAE.

• Please show isotype control staining for Figure 3G, T-bet staining.

This has been added.

• Page 4: Please remove “…. GM-CSF dependent pathogenic function”. There is no indication from this study if GM-CSF is necessary for EAE in this particular setting.

This has been modified as requested to:-

“We conclude that encephalitogenicity does not require T-bet and that this might reflect maintained GM-CSF and/or enhanced IL-17 production in its absence”.

and

“We therefore asked whether T-bet$^{-/-}$ T cells exposed to Th1-promoting conditions (IL-12 and IL-18) would maintain GM-CSF production and pathogenic function”.

Reviewer: 3

Title: the authors analysed the role of T-bet in only one model of autoimmune disorder, the title need to be changed.

We have added “CNS” into the Title.

Fig. 1B: has the cytokine production been induced by antigen specific stimulation? Or polyclonal stimulation?

As stated in the legend for Fig 1, this was pMOG-driven.

As expected, the authors analyse the ability of CD4+ T cells to produce IL-17 and IFN-g. I’m wondering if other type of lymphocytes are present in CNS at the peak of the disease, i.e CD8 cells. Moreover, if so, are they able to produce IFN-g?

We have assessed $CD8^+$ cells in previous work (Front. Immun. 2:17). We see very few $CD8^+$ cells in the WT CNS during EAE, but some of these can make IFN-γ. Although other had reported pathogenic function for $CD8^+$ cells, we have previously tried exhaustive to reproduce that effect, without success.

In the B6/MOG experiments performed for this study the majority of CNS cells that produced IFN-γ in response to pMOG were in the CD4 fraction and this was also the case in the T-$bet^{-/-}$ mice. Although we tried to detect pMOG in the $CD11b^-CD4^-$ population, the frequency of IFN-γ^+ cells in this fraction was lower in T-$bet^{-/-}$ mice, in line with the reduction of IFN-$^+$ cells seen in the $CD4^+$ population. Thus we have no evidence that would support the idea that a non-$CD4^+$ population acts as a compensatory source of IFN-γ in the T-$bet^{-/-}$. Of course, IFN- γ is not required for EAE, so such a compensatory effect would not easily explain the development of EAE in T-$bet^{-/-}$ mice.

Fig. 2: splenocytes from T-bet-/- mice stimulated in vitro with pMOG are characterized by high IL-17 production (panel C). This is expected because of the data shown by the authors in Fig. 1 and because of Th17 cells, induced in these mice during pMOG/CFA immunization, cannot be adequately polarized in vitro by IL-12 towards the Th1 phenotype (lack of T-bet). I do not understand why these Th17 cells are not able to migrate into the CNS. Is IL-12 suppressing their migratory functions? This point needs an explanation.

If we understand this comment correctly, the reviewer is asking why IL-17^+ cells produced following priming of T-$bet^{-/-}$ mice do not transfer disease after in vitro exposure to IL-12.

In our hands it is challenging to generate robustly pathogenic IL-17^+ following in vitro conditioning of primed lymph nodes, even from WT mice. For example, for our “IL-23-conditioning” we initially tried IL-23 alone, but found these cells to be non-pathogenic. The key component for the in vitro conditioning step appears to be IL-1. This may well boost expression of IL-23R on the T cells, thereby stabilizing

pathogenic function. IL-1 is not required for pathogenic function of WT IL-12-conditioned cells, and so was no included here (because this is not required to drive WT Th1 cells with pathogenic activity). Detailed work trying to define the necessary effects of IL-1 in EAE is ongoing.

Although T-bet$^{-/-}$ cells lacked CXCR3 expression (now discussed in the revised text), we have no evidence for an alternative expression of CCR6 on T-bet-deficient Th1 versus WT Th1 cells, the receptor reported (by some) to be involved in Th17 migration into the CNS.

Second Editorial Decision – 12 July 2013

Dear Prof. Anderton,

It is a pleasure to provisionally accept your manuscript entitled "T-bet is essential for Th1-mediated, but not Th17-mediated, CNS autoimmune disease" for publication in the European Journal of Immunology. For final acceptance, please follow the instructions below and return the requested items as soon as possible as we cannot process your manuscript further until all items (copyright forms etc.) are dealt with.

Please note that EJI articles are now published online a few days after final acceptance (see Accepted Articles: http://onlinelibrary.wiley.com/journal/10.1002/(ISSN)1521-4141/accepted). The files used for the Accepted Articles are the final files and information supplied by you in Manuscript Central. You should therefore check that all the information (including author names) is correct as changes will NOT be permitted until the proofs stage.

We look forward to hearing from you and thank you for submitting your manuscript to the European Journal of Immunology.

Yours sincerely,
Karen Chu

On behalf of Prof. Rikard Holmdahl

Dr. Karen Chu
Editorial Office
European Journal of Immunology
e-mail: ejied@wiley.com
